# Biophysical evaluation to categorize pathogenicity of cancer-predisposing mutations identified in the BARD1 BRCT domain†

**DOI:** 10.1039/c8ra06524a

**Published:** 2018-10-03

**Authors:** Rajan Kumar Choudhary, M. Quadir Siddiqui, Nikhil Gadewal, Nachimuthu Senthil Kumar, Ekaterina S. Kuligina, Ashok K. Varma

**Affiliations:** Advanced Centre for Treatment, Research and Education in Cancer Kharghar Navi Mumbai Maharashtra 410 210 India avarma@actrec.gov.in +91-22-2740 5085 +91-22-2740 5112; Homi Bhabha National Institute, Training School Complex Anushaktinagar Mumbai - 400 094 India; Department of Biotechnology, Mizoram University (A Central University) Aizawl – 796 004 Mizoram India; Laboratory of Molecular Oncology, Department of Tumor Growth Biology, N.N. Petrov Institute of Oncology RU-197758, Pesochny-2 St.-Petersburg Russia; Department of Biological Chemistry, The Alexander Silberman Institute of Life Sciences, Edmond J. Safra Campus, The Hebrew University of Jerusalem Jerusalem 91904 Israel; University of Nebraska Medical Centre Omaha NE USA

## Abstract

The BRCT domain of BARD1 (BARD1 BRCT) is involved in many cellular processes such as DNA damage repair (DDR) and cell-cycle checkpoint regulation. BARD1 BRCT performs tumor suppressor function by recruiting BRCA1 at DNA damage site *via* interactions with other DNA damage repair (DDR) proteins. Considering the importance of the BRCT domain in genomic integrity, we decided to evaluate reported mutations of BARD1 BRCT Cys645Arg, Val695Leu, and Ser761Asn for their pathogenicity. To explore the effect of the mutation on the structure and function, BARD1 BRCT wild-type proteins and the mutant proteins were studied using different biochemical, biophysical and *in silico* techniques. Comparative fluorescence, circular dichroism (CD) spectroscopy and limited proteolysis studies demonstrate the well-folded structural conformation of wild-type and mutant proteins. However, thermal and chemical denaturation studies revealed similarity in the folding pattern of BARD1 BRCT wild-type and Cys645Arg mutant proteins, whereas there was a significant loss in the thermodynamic stability of Val695Leu and Ser761Asn mutants. Molecular dynamics (MD) simulation studies on wild-type and mutant protein structures indicate the loss in structural integrity of mutants compared with the wild-type protein.

## Abstract 2

The BRCT domain of BARD1 (BARD1 BRCT) is involved in many cellular processes such as DNA damage repair (DDR) and cell-cycle checkpoint regulation.

## Graphical abstract

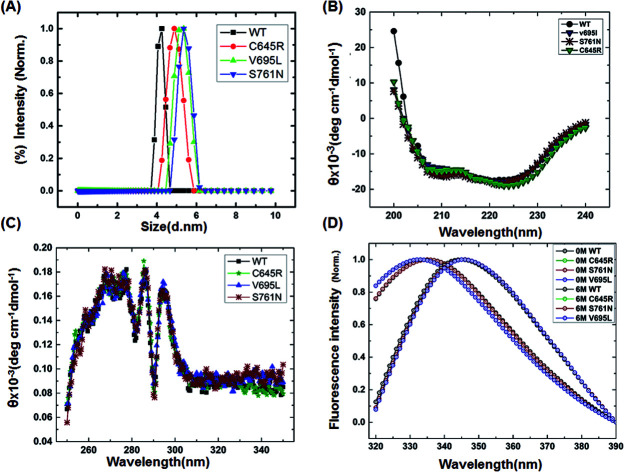

## 1 Introduction

BARD1 (BRCA1-associated Ring Domain protein) has been identified as an N-terminal binding partner of tumor suppressor protein BRCA1 (breast cancer-associated gene product 1).^1–3^ BRCA1/BARD1 N-terminal RING–RING domain heterodimer is an E3 ubiquitin ligase complex and has an indispensable role in tumor suppression.^3–8^ Mutations in the RING domain of BRCA1 that abolish the interactions with BARD1 have been found to be associated with breast and ovarian cancers, thus establishing BARD1 as a tumor suppressor.^5,9^ Up-regulated level of BARD1 after DNA damage, hormone signaling, hypoxic condition,^10^ and induction of apoptosis by activation of caspase 3 in a p53-mediated fashion demonstrated the involvement of BARD1 in tumor suppression.^11,12^ In several breast, ovarian and uterine tumor cases, BARD1 was found to be either mutated or truncated.^13–15^ The BARD1 protein comprises 777 amino acids and different functional domains including one N-terminal ring domain region, one ankyrin repeat domain^16^ and two tandem BRCT repeat domains structurally similar to the BRCA1 BRCT domain.^17^

BRCT repeat domains are present in different DNA repair proteins, such as MDC1, BRCA1, BARD1, XRCC1, and 53BP1, and dynamically participate in DNA damage repair mechanism.^18–24^ BARD1 BRCT also has two conserved binding pockets, P_1_ (hydrophilic pocket) and P_2_ (hydrophobic pocket), for interaction with DNA damage repair protein in a phospho-dependent manner.^25^ The role of BARD1 BRCT in DNA damage repair is crucial for early recruitment of BRCA1 to the DNA damage site.^26^ Three cancer-predisposing mutations Cys645Arg, Val695Leu, and Ser761Asn are reported within the BARD1 BRCT domain^15,27^ in the breast, ovarian and uterine cancers.^15,28,29^ Recent studies have established that BARD1 BRCT is required for early recruitment of BRCA1 at the DNA damage site *via* directly interacting with PAR through the residues Cys645 and Val695.^26^ In the crystal structure of BARD1 BRCT,^30^ Ser761Asn mutation is located in very close proximity to the conserved P_2_-binding pocket that may interfere with phosphopeptide binding at P_2_ pocket.^30^ A set of *in silico* pathogenicity prediction tools such as Align-GVGD^31^ (Align-Grantham variation (GV) and Grantham deviation (GD)), I-mutant 3.0 (ref. 32) (prediction of protein stability changes upon single point mutation), and SNP&GO^33^ (single amino acid polymorphisms using GO terms) have been used for the prediction of pathogenicity of mutations. Furthermore, to understand the abrogative effects of mutations, a combinatorial approach of biochemical, biophysical and *in silico* techniques were employed. This study will also help in understanding the role of BARD1 BRCT in DNA damage repair and future development of small molecule inhibitors, which can modulate or neutralize the pathogenic effects of mutations.

## 2 Results

### 2.1 Pathogenicity characterization of disease-associated mutants

**Fig. 1:**
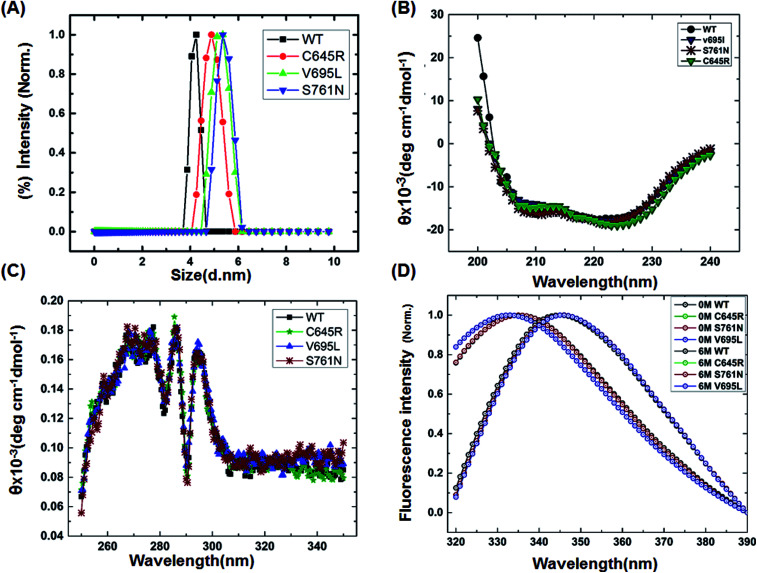
Structural characterization of BARD1 BRCT wild-type and mutant proteins. (A) Dynamic light-scattering profile of the BARD1 BRCT wild-type and mutant proteins. (B) Secondary structure analysis of BARD1 BRCT wild-type and mutant proteins using far-UV, CD spectroscopy. (C) Tertiary structure characterization of BARD 1BRCT wild-type and mutant proteins using near-UV, CD spectroscopy. (D) Chemical denaturation of BARD1 BRCT wild-type and mutant proteins using GuHcl.

The BARD1 BRCT Cys645Arg, Val695Leu, and Ser761Asn mutations are identified as cancer predisposing in nature (Fig. 1A, ESI†), and significantly affect the tumor-suppressor functions of the BARD1.^15^ The ΔΔ*G*° value predicted by I-mutant 3.0 (ref. 32) is tabulated in Table 1 (ESI†). It is observed that ΔΔ*G*° value predicted for BARD1 BRCT Cys645Arg mutant protein is −0.29 kcal mol^−1^, which is smaller than the set threshold of −0.5 kcal mol^−1^. Therefore, Cys645Arg mutation is classified as neutral without any pathogenicity. However, ΔΔ*G*° values for Val695Leu and Ser761Asn mutants are −1.82 kcal mol^−1^ and −0.54 kcal mol^−1^, respectively. Therefore, it can be predicted that Val695Leu and Ser761Asn mutations in the BARD1 BRCT domain may be pathogenic. The prediction is also supported by SNP&GO,^33^ which indicates that except Cys645Arg substitution, Val695Leu and Ser761Asn substitutions have chances of disease predisposition (Table 1, ESI†). We also performed pathogenicity analysis Align-GVGD^31^ to characterize these mutants. Align-GVGD classifies BARD1 BRCT Cys645Arg mutant protein as most likely to be pathogenic and scores this protein as class 65, while Ser761Asn is scored as class 45 and Val695Leu is scored as class 25, classifying as less pathogenic (Table 1).

**Table 1:** *In silico* prediction of pathogenicity of BARD1 BRCT mutants. Comparative prediction output from different prediction servers indicates that mutation may have structural and functional effects on the BARD1 BRCT domain

Mutation	I-mutant prediction	−ΔΔ*G*°	SNP&GO prediction	Align-GVGD
Cys645Arg	Decreased stability	−0.29 kcal mol^−1^	Neutral	Deleterious (class C65)
Val695Leu	Decreased stability	−1.82 kcal mol^−1^	Disease	Neutral (class C35)
Ser761Asn	Decreased stability	−0.54 kcal mol^−1^	Disease	Deleterious (class C65)

### 2.2 Oligomeric behavior of BARD1 BRCT mutants

**Fig. 2:**
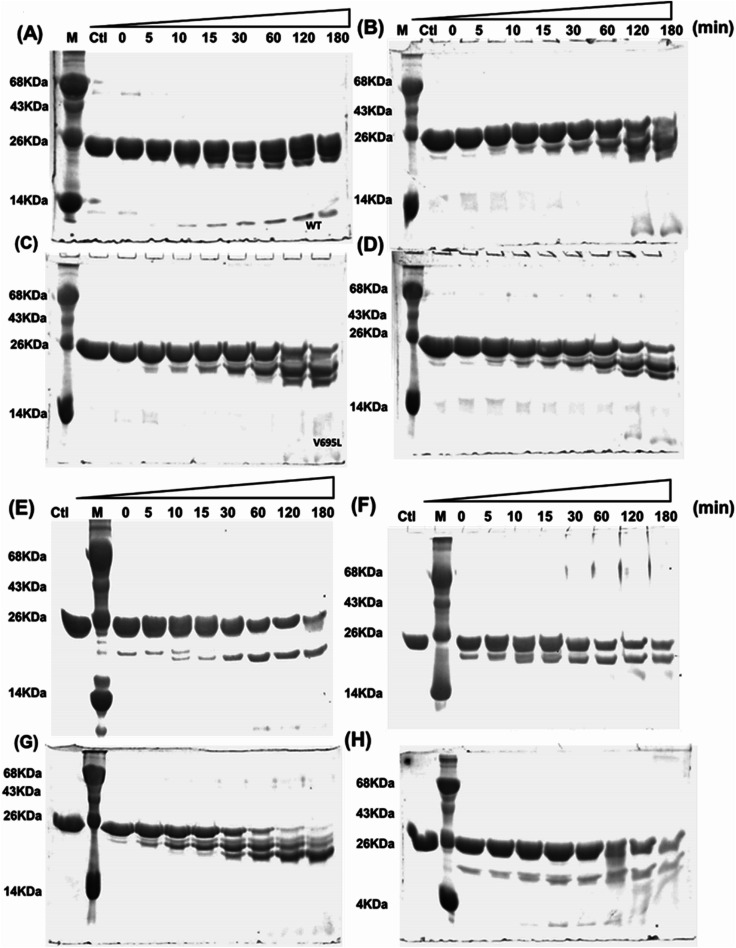
Limited proteolysis of BARD1 BRCT wild-type and mutant proteins. (A–D) are trypsin digestion profiles of wild-type, Cys645Arg, Val695Leu and Ser761Asn mutant, respectively. (E–H) are chymotrypsin digestion profiles of wild-type, Cys645Arg, Val695Leu, and Ser761Asn mutant, respectively. Control (Ctl) shows untreated trypsin and chymotrypsin sample of wild-type and mutant proteins.

To characterize the effect of the mutation on BARD1 BRCT, wild-type and mutant proteins were purified using Ni-NTA affinity chromatography (Fig. 1B, ESI†). The size exclusion chromatography profiles of BARD1 BRCT wild-type, and Cys645Arg, Val695Leu, and Ser761Asn mutants are shown in Fig. 1C (ESI†). As reported earlier, wild-type BARD1 BRCT is monomeric in nature,^34^ and comparative gel filtration elution profile of BARD1 BRCT wild-type and mutant proteins indicated that all the proteins elute at same elution volume, suggesting that mutations do not affect the monomeric property of the BARD1 BRCT protein (Fig. 1C, ESI†). DLS studies revealed that BARD1 BRCT wild-type and the mutants Cys645Arg, Val695Leu, and Ser761Asn had hydrodynamic radii in the range of 2.2–2.7 nm. These radii are comparable with the Stoke's radii of 2.4 nm obtained from the gel filtration chromatography calibrated against the known standards. The experimental hydrodynamic diameter obtained from DLS for wild-type was 4.2 ± 0.19 nm and for mutants, the hydrodynamic diameters were 4.88 ± 0.32 nm for Cys645Arg, 5.12 nm ± 0.67 nm for Val695Leu and 5.35 ± 0.43 nm for Ser761Asn, which conclude that BARD1 BRCT and the mutants are monomeric in nature (Fig. 1A). This inference is also supported by SDS-PAGE glutaraldehyde cross-linking profiles of wild-type and mutant proteins. As there was no sign of any oligomeric species observed for wild-type and mutant proteins, mutations did not change the monomeric property of BARD1 BRCT (Fig. 2, ESI†).^25,30^

### 2.3 Mutants have structures similar to wild-type

Far- and near-UV range CD (circular dichroism spectroscopy) spectra for BARD1 BRCT wild-type and Cys645Arg, Val695Leu, and Ser761Asn mutants are shown in Fig. 1B and C. The ellipticity minima at *λ* = 222 nm and *λ* = 208 nm indicate the presence of predominantly α-helical structures. The ellipticity maximum rise at *λ* = 218 nm indicates the presence of β-strands, and both these features are in good agreement with the crystal structure of BARD1 BRCT (PDB ID: 2NTE, Fig. 1A, ESI†). The far-UV spectra of wild-type and mutant proteins do not show any large change in the ellipticity pattern, indicating that mutations are not drastically changing the secondary structure of the protein. Near-UV spectra of BARD1 BRCT wild-type and mutant proteins are shown in Fig. 1C. All the proteins including wild-type and mutant proteins show a characteristic peak at *λ* = 295 nm and a plateau from *λ* = 260–280 (cumulative signal for Trp, Phe, Tyr amino acids), which is an indication of the hydrophobic micro-environment around the aromatic residues in the structure (Fig. 1C). Tryptophan fluorescence was also measured in the presence of GuHcl to probe the compactness of tertiary structural differences between BARD1 BRCT wild-type and mutant proteins. Emission maxima recorded for native BARD1 BRCT wild-type, and Cys645Arg, Val695Leu, and Ser761Asn mutant proteins were at *λ* = 332 nm, 333 nm, 333 nm and 335 nm, respectively. However, a shift in the emission maxima is highest for the Ser761Asn mutant, which is very close to that of the Trp762 fluorophore. BARD1 BRCT wild-type and mutant proteins show emission maxima at *λ* = 348 nm for the unfolded state at 6 M GuHcl (Fig. 1D). Furthermore, to explore the effect of the mutation on BARD1 BRCT tertiary structure, limited proteolysis was performed, and the results are shown in Fig. 2. Limited proteolysis profile of BARD1 BRCT wild-type and mutant proteins show similar resistivity profiles for trypsin (Fig. 2A–D) and chymotrypsin digestion, unravelling the facts that BARD1 BRCT wild-type is a stable domain and the mutations brought neither large tertiary structural changes nor overall packing of mutant proteins (Fig. 2E–H).

### 2.4 Thermodynamics and unfolding

**Fig. 3:**
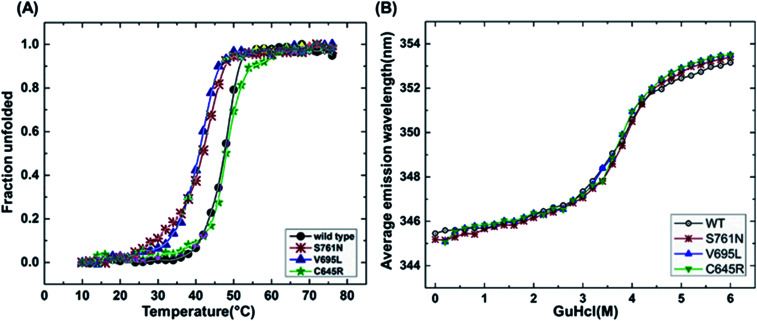
Thermal and chemical denaturation profiles of BARD1 BRCT wild-type and mutant proteins. (A) Comparative overlay of fraction unfolded of BARD1 BRCT wild-type and Cys645Arg, Val695Leu and Ser761Asn mutants. (B) Chemical denaturation profiles of BARD1 BRCT wild-type and Cys645Arg, Val695Leu and Ser761Asn mutants.

**Fig. 4:**
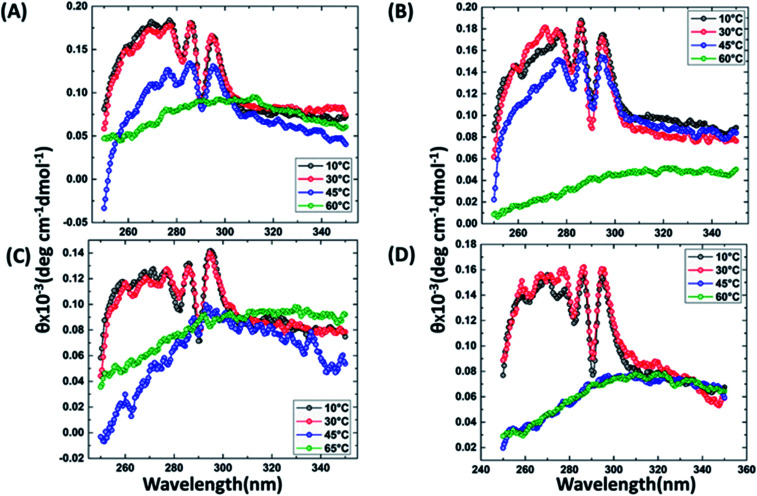
Comparative thermal denaturation profile of BARD1 BRCT wild-type and mutant proteins in near-UV range. Thermal denaturation profile of BARD1 BRCT (A) wild-type, (B) Cys645Arg, (C) Ser761Asn and (D) Val695Leu.

It has been reported that BRCT domains unfold *via* intermediate molten globule formation.^25,34–37^ Thermal denaturation profiles of BARD1 BRCT wild-type and Cys645Arg, Val695Leu and Ser761Asn mutants are shown in Fig. 3A. It is observed that BARD1 BRCT wild-type and Cys645Arg have higher thermal stability compared with Val695Leu and Ser761Asn mutants. BARD1 BRCT wild-type and mutant proteins unfold *via* a two-state transition. However, Ser761Asn mutant protein lost the cooperativity and *T*_m_ significantly (Fig. 3A). The *T*_m_'s values calculated for BARD1 BRCT wild-type, Cys645Arg, Val695Leu and Ser761Asn assuming a two-state transition model^34,38,39^ were 47.7 ± 0.85 °C, 47.6 ± 0.65 °C, 42.3 ± 0.78 °C, and 41.1 ± 0.42 °C, respectively. Δ*G*_N-D_ calculated for BARD1 BRCT wild-type, Cys645Arg, Val695Leu, and Ser761Asn mutant proteins were 9.8 ± 0.39 kcal mol^−1^, 9.6 ± 0.11 kcal mol^−1^, 7.4 ± 0.69 kcal mol^−1^ and 7.1 ± 0.12 kcal mol^−1^, respectively (Fig. 3A). The chemical denaturation profiles of BARD1 BRCT wild-type and mutant proteins at 10 °C are shown in Fig. 3B. The chemical denaturation profile shows that BARD1 BRCT wild-type and mutant proteins completely unfold at a concentration of 6 M GuHcl, following a three-state unfolding pathway with an intermediate formation at 2.2 M GuHcl (Fig. 3B). The Δ*G*_N-I-D_ values calculated for BARD1 BRCT wild-type, Cys645Arg, Val695Leu, and Ser761Asn mutant proteins were 6.94 ± 0.23 kcal mol^−1^, 6.28 ± 0.82 kcal mol^−1^, 6.2 ± 0.45 kcal mol^−1^, and 6.11 ± 0.18 kcal mol^−1^, respectively. The near-UV spectra for wild-type and mutant proteins are shown in Fig. 4A–D. At 10 °C, BARD1 BRCT wild-type and mutant proteins are present in fully folded native conformation. However, with the increase in temperature to 45 °C, BARD1 BRCT Val695Leu and Ser761Asn mutant proteins show reduced CD signal compared with the wild-type protein (Fig. 4C and D), which is an indication of significant loss in the tertiary structure of mutant proteins (Fig. 4A–D). However, BARD1 BRCT wild-type and the BARD1 BRCT Cys645Arg mutant partially lose their tertiary structure at 45 °C (Fig. 4A and B), providing an indication that Ser761Asn and Val695Leu mutant proteins have lost their thermal stability compared with the BARD1 BRCT wild-type and Cys645Arg mutant protein.

### 2.5 Variable temperature fluorescence spectroscopy

Fluorescence spectra were recorded from 15 °C to 60 °C at 3 °C intervals, for BARD1 BRCT wild-type and the mutant proteins (Fig. 3, ESI†). The high fluorescence emission intensity recorded at 15 °C indicates complete burial of tryptophan in the hydrophobic core of the protein.^40^ However, as the temperature increases up to 30 °C, the loss in fluorescence intensity for wild-type and mutant proteins suggests the restricted opening of the compact structure due to partial exposure of tryptophan to the polar environment. Furthermore, no significant decrease in the fluorescence intensity was observed at 45 °C for wild-type and Cys645Arg mutant proteins. However, at 45 °C, Ser761Asn and Val695Leu mutants show the increase in fluorescence intensity and blue-shifting in emission maxima as compared with wild-type and Cys645Arg mutant proteins. At 50 °C, the fluorescence intensity was maximum for wild-type and mutant proteins with blue-shift in emission maxima, indicating burial of tryptophan fluorophores in the hydrophobic core of the protein for the formation of a molten globule structure. The higher fluorescence intensity and blue-shift at 45 °C for Val695Leu and Ser761Asn mutant indicate molten globule formation at lower temperature compared with Cys645Arg and wild-type protein (Fig. 3, ESI†). The changes in the emission maximum of wild-type and mutant proteins are tabulated in Table 2 (ESI†).

### 2.6 Molecular dynamics simulation analysis of BARD1 BRCT and mutants

**Fig. 5:**
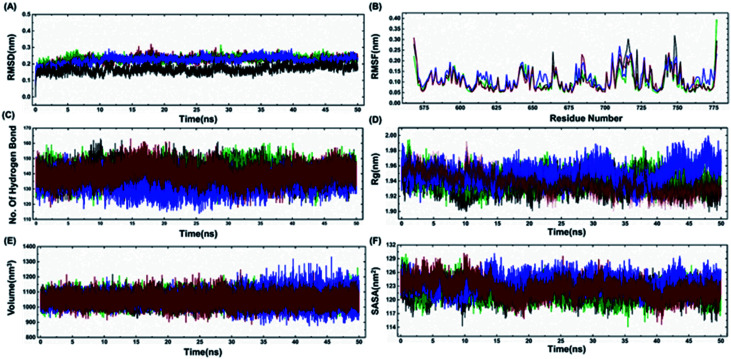
Comparative representation of MDS trajectory for wild-type and mutant proteins. Comparative profile of (A) RMSD, (B) RMSF, (C) number of hydrogen bonds, (D) radius of gyration, (E) volume (nm^3^), and (F) SASA (nm^2^) of wild-type, Cys645Arg, Val695Leu and Ser761Asn (black color represents wild-type, green color indicates Cys645Arg profile, blue color indicates Val695Leu and red color line represents Ser761Asn mutant protein-structure profile).

The model structures of all three cancer predisposing mutants Cys645Arg, Val695Leu and Ser761Asn reported in the BARD1 BRCT region were prepared *via* introducing mutations in the amino acid sequences of the wild-type protein using a Swiss PDB viewer.^41^ These structures were subjected to molecular dynamics simulation (MD) under periodic boundary conditions. After the equilibration period, the comparative RMSD (root-mean-square deviation) profile of BARD1 BRCT wild-type and mutant proteins indicated that the wild-type structure shows stable RMSD and trajectory compared with mutants throughout the simulation period. BARD1 BRCT Val695Leu mutant shows RMSD fluctuation in profiles similar to Cys645Arg and Ser761Asn mutant structures (Fig. 5A). The comparative RMSF (root-mean-square fluctuation) analysis indicates that there is a local structural change in mutant proteins compared with the BARD1 BRCT wild-type protein (Fig. 5B). The weak intramolecular hydrogen bonding profile indicates that except Cys645Arg mutation, BARD1 BRCT Val695Leu and Ser761Asn mutations have less number of hydrogen bonds as compared with the wild-type protein (Fig. 5C and 4, ESI†). Fluctuation in the radius of gyration (*R*_g_) provides a measure of molecular compactness of the dynamic system (Fig. 5D). The *R*_g_ fluctuation was found to be higher in case of BARD1 BRCT Val695Leu mutant structure, which has a positive correlation with the reduced number of intra-molecular hydrogen bonds (Fig. 5C and 4, ESI†). Comparative volume change observed for BARD1 BRCT wild-type and mutant protein structures clearly indicate that BARD1 BRCT Val695Leu substitution has destabilized the hydrophobic core in the mutant protein structure (Fig. 5E). The solvent accessible surface area (SASA) calculated after 50 ns of MD production run indicates that BARD1 BRCT Val695Leu mutant protein shows more exposed solvent accessible surface area compared with other mutants and wild-type protein (Fig. 5F) (Table 2).

**Table 2:** Results obtained from fluorescence spectroscopy thermal denaturation showing change in emission maximum at different temperatures

	WT	Cys645Arg	Val695Leu	Ser761Asn
15 °C	332 nm	333 nm	333 nm	335 nm
30 °C	330 nm	333 nm	333 nm	333 nm
45 °C	328 nm	331 nm	331 nm	331 nm
50 °C	329 nm	330 nm	329 nm	329 nm

### 2.7 Identifying the conformational fluctuation by principal component analysis (PCA)

**Fig. 6:**
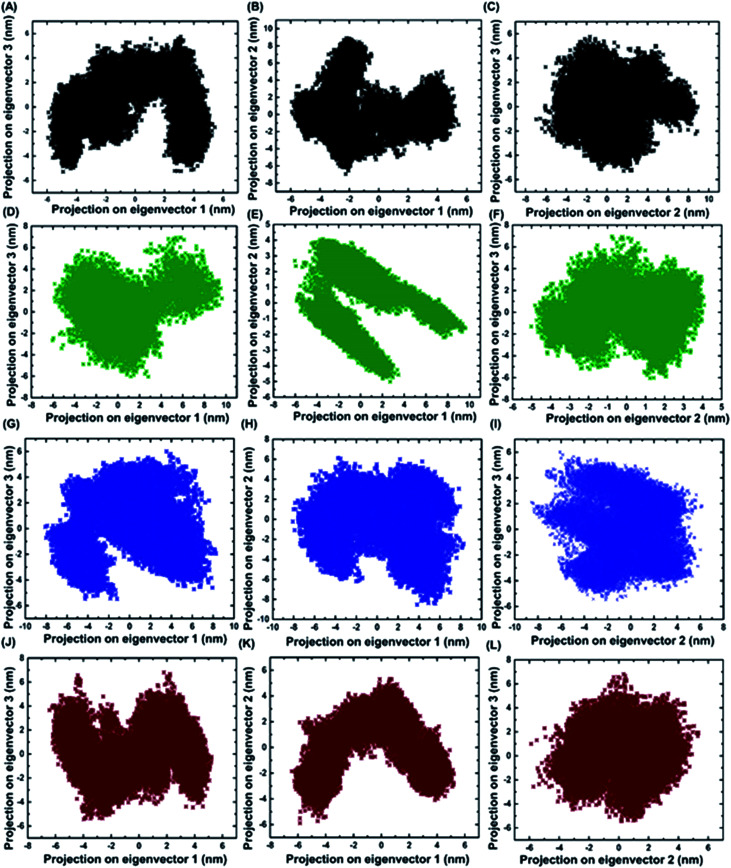
Projections of first three eigenvectors for wild-type and mutant proteins. (A) Projection of eigenvector 3 on 1, (B) 2 on 1 and (C) 3 on 2 for wild-type. (D) Projection of eigenvector 3 on 1, (E) 2 on 1 and (F) 3 on 2 for Cys645Arg. (G) Projection of eigenvector 3 on 1, (H) 2 on 1 and (I) 3 on 2 for Val695Leu. (J) Projection of eigenvector 3 on 1, (K) 2 on 1 and (L) 3 on 2 for Ser761Asn.

**Fig. 7:**
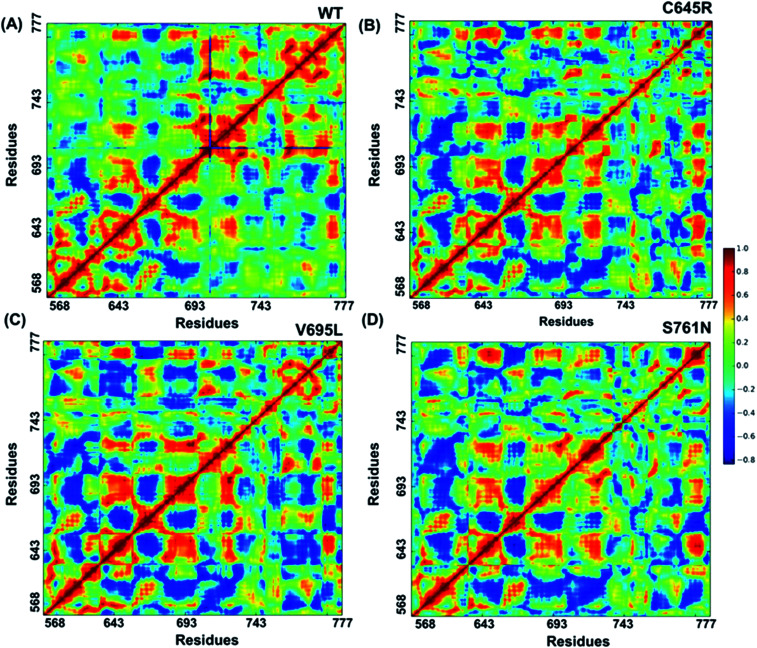
Cross-correlations for PCA of wild-type and mutant proteins. (A) Cross-correlation for PCA of wild-type, (B) Cys645Arg, (C) Val695Leu and (D) Ser761Asn mutant proteins.

Principal component analysis was performed for BARD1 BRCT wild-type and mutant protein trajectories after diagonalization of the covariance matrix to obtain eigenvalues and eigenvectors (Fig. 6). The trace of covariance matrix were 64.97 nm^2^, 58.49 nm^2^, 57.22 nm^2^, and 53.89 nm^2^ for Val695Leu, Ser761Asn, Cys645Arg and wild-type proteins, respectively. Val695Leu shows the highest trace of covariance matrix, implying greater structural flexibility compared with wild-type, Ser761Asn and Cys645Arg mutant proteins (Table 3, ESI†). The first three eigenvectors having the highest corresponding eigenvalues cover 90% of atomic motion attained by wild-type and mutant proteins. Fig. 5 (ESI†) shows that eigenvalue change for Val695Leu mutant for the first two eigenvectors is the highest as compared with wild-type and mutant proteins. Fig. 6A–L show the projection of trajectory on to first three eigenvectors for wild-type and mutant proteins. BARD1 Val695Leu protein structure shows the highest periodic transition in tertiary structure when projected on eigenvectors 2 and 1, 3 and 1 and 3 and 2 compared with wild-type and other mutant protein structures, demonstrating the higher structural flexibility (Fig. 6G–I). The wild-type (Fig. 6A–C), Cys645Arg (Fig. 6D–F) and Ser761Asn (Fig. 6J–L) show at least two different tertiary structure clusters. However, Val695Leu shows three different tertiary structural clusters, indicating higher structural dynamics (Fig. 6I). Fig. 6 (ESI†) shows displacements of components of the wild-type and mutant proteins for the eigenvectors 1 and 2. The projection on first two eigenvectors of the residues for wild-type shows fluctuation in the c-terminal loop regions and the connecting loop between N-terminal BRCT and C-terminal BRCT domain, which are consistent with RMSF structure obtained (Fig. 7A, ESI†). In case of Cys645Arg mutant, projection on eigenvectors 1 and 2 of the residue indicates high fluctuation at 568–610 and 740–750 amino acid residue regions, which is also reflected in the RMSF structure (Fig. 6C and 7B, ESI†). Furthermore, projection on eigenvectors 1 and 2 in the structures of residues for BARD1 BRCT Val695Leu mutant protein specifies that the mutational effect on the residual fluctuation is not localized (Fig. 6E and F, ESI†). The RMSF structure for BARD1 BRCT Val695Leu also indicates that mutation has affected the structure at the global level (Fig. 7C, ESI†). Projection on eigenvectors 1 and 2 of residues for BARD1 BRCT Ser761Asnmutant protein structure reveals fluctuation in connecting loops at C-terminal BRCT (Fig. 6G and H and 1A, ESI†), which shows consistency with the RMSF structure (Fig. 7D, ESI†) (Table 3).

**Table 3:** Trace of covariance matrix obtained for BARD1 BRCT wild-type and mutant proteins after PCA

BARD1 BRCT and mutants	Trace of covariance matrix
WT	53.89 nm^2^
Cys645Arg	57.22 nm^2^
Val695Leu	64.97 nm^2^
Ser761Asn	58.49 nm^2^

### 2.8 Covariance analysis

Cross-correlation matrixes of fluctuations for C-alpha atoms around their mean positions for wild-type and mutant proteins are represented in Fig. 7. The BARD1 BRCT wild-type protein shows a positive correlation within the N-terminal and C-terminal residues, but most of the N-terminal residues show a negative correlation with the C-terminal residues (Fig. 7A). When compared with wild-type, Cys645Arg and other mutant proteins show loss of positive residual correlation within N- and C-terminal BRCT residues (Fig. 7B–D). However, BARD1 BRCT Val695Leu protein structure shows a moderate decrease in the negative correlation within the N-terminal BRCT residues and increased positive correlation in atomic motion in C-terminal BRCT residues as compared with Cys645Arg and Ser761Asn (Fig. 7C). The BARD1 BRCT Ser761Asn mutant structure also shows localized changes in the positive correlation in the N-terminal (610–650 aa) and C-terminal (760–770 aa) BRCT region (Fig. 7D). These transformations reflect the difference in the internal dynamics of mutants in comparison with the wild-type structure.

### 2.9 Secondary structure characterization by DSSP

**Fig. 8:**
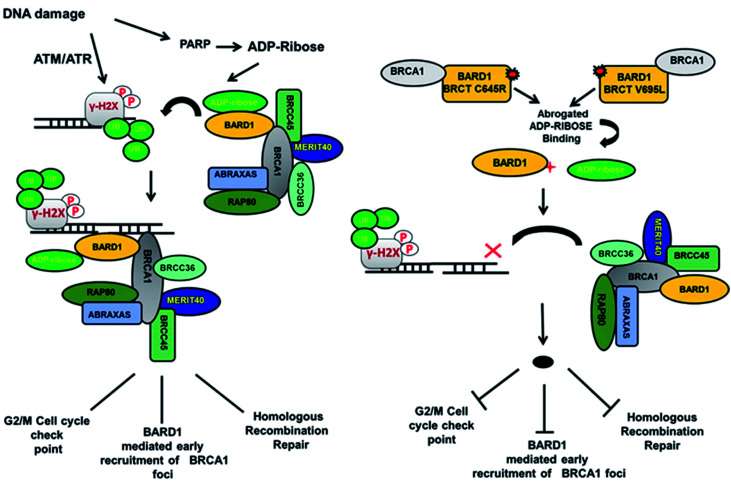
Model representing the consequence of Cys645Arg and Val695Leu mutations on BARD1 BRCT function and DNA damage repair.

Comparative DSSP (dictionary of secondary structure of proteins) characterization of secondary structure has been presented in Fig. 8A–D (ESI†). It may be observed that the mutations have a local effect on the secondary structure of the BARD1 BRCT wild-type protein. Mutations Cys645Arg and Ser761Asn are present on the surface of BARD1 BRCT, while the mutation Val695Leu is present in the hydrophobic core of the protein. Though Cys645 substitution to arginine changes the chemical character of the amino acid from hydrophobic to basic, because of its presence in a loop region of BARD1 BRCT, it has no effect on the secondary structure of the protein. This observation is consistent with the results of CD spectroscopic analysis (Fig. 8A–D, ESI†). The BARD1 BRCT Val 695 Leu substitution is presented in the α′2 helix (Fig. 1A, ESI†), and the structure also shows no significant change in the structure as compared with the wild-type in CD spectroscopy as well as DSSP analysis. This intriguing change can be explained on the basis of the α-helix propensity of the amino acids.^42^ The BARD1 BRCT Ser761Asn mutation is present in the α′3 helix of BARD1 BRCT (Fig. 1A, ESI†) and does not show a drastic secondary structural change in the DSSP analysis. The propensity of alpha-helix formation by serine is higher than that by asparagine, which creates a possibility of losing the secondary structure. However, in the presence of strong neighboring α-helix former amino acids, the balance between enthalpic effects and conformational entropy is compensated.^42^ Therefore, even after serine-to-asparagine substitution, we did not observe any drastic secondary structure transformation in the CD spectroscopy as well as DSSP analysis.

### 2.10 Intra-molecular interactions analysis

BARD1 Cys645 in the wild-type structure was found to have non-covalent interactions with Leu592 and Leu570. However, in the molecular model of BARD1 BRCT Cys645Arg mutant structure, Arg645 shows loss of non-covalent interactions with Leu592 and gain of hydrogen bonding interactions with Leu592 (Fig. 9A and B, ESI†). Similarly, in BARD1 BRCT wild-type, Val695 shows a hydrogen-bonding interaction with Gly698, Thr696, Ile692, and Leu691. Moreover, Val695 is also involved in the non-covalent interaction with Phe577, Phe763, Gly698, Lys693, and Ala697. In Val695Leu mutant, Leu695 shows one less hydrogen bonding interaction by limiting its interaction with Gly699, Gly700 andLeu691. Leu695 in mutant structure shows non-hydrogen bonding interaction similar to wild-type structure (Fig. 9C and D, ESI†). Ser761 in BARD1 BRCT wild-type is found to have very few interacting residues in its neighborhood. It forms a hydrogen bond with Ile764 and non-covalent interaction with Pro759, Asp741, and Phe763 (Fig. 9E, ESI†). Interestingly, in mutant structure, Asn761 forms a hydrogen bond with Asp741 and non-covalent interaction with Pro759 and Phe763 (Fig. 9F, ESI†). Loss of hydrogen bonding interaction with Ile764, which is an important residue suspected to interact with the phosphopeptide _s_P-x-x-t-F, may lead to alteration of hydrophobic pocket architecture, which can lead to loss of phosphopeptide docking site for DNA damage repair proteins.

## 3 Discussion

*In silico*, biophysical and biochemical tools were used to demonstrate the pathogenicity of BARD1 Cys645Arg, Val695Leu, and Ser761Asn mutations. *In silico* prediction from I-mutant 3.0, SNP&GO and Align-GVGD servers clearly indicated that these mutations might be pathogenic in nature. Gel filtration, DLS, and chemical crosslinking profiles of the protein suggest that the monomeric property of BARD1 BRCT domain is not affected by mutations. The secondary structure investigation using CD spectroscopy indicates that these mutations do not drastically alter the secondary and tertiary structural composition of the BARD1 BRCT domain. Thermal denaturation studies using CD spectroscopy suggests that BARD1 BRCT wild-type and BARD1, Ser761Asn, Cys645Arg and Val695Leu mutants unfold *via* a two-state pathway. The *T*_m_ values suggest that Val695Leu and Ser761Asn mutants lose their thermodynamic stability. The tertiary structure characterization using fluorescence spectroscopy suggests that BARD1 BRCT wild-type and mutant proteins unfold *via* a three-state pathway. Moreover, the results from chemical denaturation are consistent with the results obtained from thermal denaturation by fluorescence spectroscopy.

According to molecular dynamics simulation, all the mutants show higher RMSD value as compared with the wild-type structure. The RMSF profile for wild-type and mutant structure indicates that the mutation has brought only local changes in the RMSF values. Comparative hydrogen bonding profile indicates that the BARD1 Val695Leu structure significantly loses hydrogen bonds as compared with the wild-type structure. Val695 residue is buried in the hydrophobic core of the BARD1 BRCT structure. Substitution of valine into leucine can lead to the addition of extra –CH_2_– groups, which can bring entropic changes in the hydrophobic core of the protein, and in turn result in an increase in the volume and the solvent accessible area. Intra-molecular hydrogen bonding is one of the major forces holding the secondary structure of the protein, and the reduced number of H-bonds in the BARD1 BRCT Val695Leu and Ser761Asn mutant structures could be the possible reason behind the loss of compactness and thermodynamic stability.

Comparative circular dichroism spectroscopy profile and DSSP analysis suggest no significant change in the secondary structure of the mutant proteins. No drastic changes were observed in the structure of BARD1 Cys645Arg mutant due to its location in the BARD1 BRCT structure. Its presence in the random coil region may lead to high local conformational entropy and different rotameric states of arginine^43^ without affecting the secondary structure of BARD1 BRCT. Moreover, no large changes are observed in the secondary structure in valine and leucine mutants because of the frequent existence of leucine in α-helix and it is a strong helix former than valine.^44^ It has been reported that due to conformational entropy, leucine is thermodynamically more favored in α-helix than valine.^42,45,46^ However, in BARD1 Ser761Asn substitution, a small polar amino acid has been converted into asparagine, which has a comparatively large side chain. Serine has a slightly higher α-helix propensity compared with asparagine. Therefore, a small local change can be expected, but capturing this minute change is beyond the sensitivity of CD spectroscopy.^42^ The possibility of losing the secondary structure in BARD1 Ser761Asn mutant is also insignificant because of the presence of strong α-helix forming neighboring amino acids.^42^

Loss of hydrogen bond can lead to a loss in structural compactness, which has been revealed in the PCA. BARD1 Val695Leu mutant shows high atomic motion and periodic jump in the tertiary structure when projected on the first three eigenvectors. Cross-correlation of PCA for wild-type and mutant proteins indicates a significant change in the correlated atomic motion of the mutant as compared with the wild-type protein structure. Intra-molecular interaction analysis performed over the minimum energy structures signifies that BARD1 Val695Leu has lost hydrogen bonding interaction. BARD1 Ser761Asn shows the loss in hydrogen bonding with Ile764, which is a critical residue in the hydrophobic peptide-binding pocket, and may lead to the structural alterations in phosphopeptide-binding pocket. The studies were performed on BRCA1 BRCT and other BRCT repeat domains containing proteins^35,47–51^ and the mutations in BARD1 BRCT lead to distinct changes in the hydrogen bonding and non-covalent interactions, which could be responsible for the loss of BARD1 BRCT-phosphopeptide and ADP-ribose binding and early recruitment of BRCA1 at the DNA damage site.^26^ Studies of changes in the hydrogen bonding and non-covalent interactions, which drives the BARD1 BRCT–ligand interaction, will thus be helpful in the development of high-affinity small-molecule BARD1 BRCT inhibitors. In conclusion, an alteration in structure and dynamics of BARD1 BRCT native structure by different mutations impairs the functionality of BARD1 BRCT as an important DNA damage repair protein (Fig. 8).

## 4 Experimental

### 4.1 Protein expression and purification of BARD1 BRCT wild-type and mutant proteins

The BARD1 BRCT (568–777) was expressed and purified as reported earlier.^25,30,34^ BARD1 BRCT mutants Cys645Arg, Val695Leu and Ser761Asn were generated by site-directed mutagenesis using mismatch forward and reverse primers. All the mutants were confirmed by DNA sequencing. The mutant proteins were expressed in *Escherichia coli BL21 (DE3)* cells grown at 37 °C until absorbance reached between 0.6 and 0.8 at 600 nm, followed by induction with 0.1 mM IPTG at 18 °C for 20 h. The His-tagged BARD1 BRCT proteins were affinity-purified in 50 mM Tris, 500 mM NaCl, 5% glycerol and 2 mM EDTA buffer (pH 7.5) supplemented with the protease inhibitors (Roche Applied Science). The protein of interest bound to Ni-NTA column was eluted by using a gradient concentration of imidazole and treated with TEV protease to remove the His tag. The mutant proteins were further purified using FPLC buffer (10 mM HEPES, 150 mM NaCl, pH 7.5) for biophysical characterization.

### 4.2 Chemical denaturation of BARD1 BRCT wild-type and mutant proteins

Chemical denaturation of BARD1 BRCT wild-type and mutant proteins Cys645Arg, Val695Leu and Ser761Asn was performed at 10 °C. By using GuHcl as a denaturant, tryptophan microenvironment was monitored using a fluorescence spectrophotometer (Horiba, USA) at excitation wavelength *λ* = 295 nm. The 10 M GuHcl (Sigma-Aldrich) stock solutions were diluted in the range of 0–6 M with 2 μM of BARD1 BRCT wild-type and mutant proteins in buffer (10 mM HEPES, 150 mM NaCl, pH 7.5) with increasing GuHcl concentration. Fluorescence emission spectra were recorded with a concentration change from *λ* = 310–400 nm and blank subtractions were performed. In chemical denaturation, a three-state model (folded (N), intermediate (I) and unfolded (U)) was used for curve fitting.^34,38,39^ Furthermore, circular dichroism spectra of BARD1 BRCT wild-type and mutant proteins were recorded using sealed quartz cuvettes of 1 mm path length using a JASCO J-815 spectropolarimeter (Jasco, Easton, MD). BARD1 BRCT wild-type and mutant proteins at concentrations of 10 μM and 40 μM were scanned in the far-UV (*λ* = 200–240 nm) and near-UV range (*λ* = 350–250 nm), respectively. An average of seven spectra at a scan speed of 20 nm min^−1^ with a resolution of 1 nm and response time of 1 s were used for representation. A standard blank correction was applied to the raw data. The results are presented as mean residual ellipticity [*θ*] (deg cm^2^ dmol^−1^).

### 4.3 Thermal denaturation

Thermal denaturation of BARD1 BRCT wild-type and mutant proteins at a concentration of 10 μM and 40 μM in buffer A causes unfolding over a temperature range of 5–75 °C. Spectra were recorded both in far-UV range (200–240 nm) and near-UV range (350–250 nm) at a scan speed of 20 nm s^−1^. The sample was heated at a rate of 1 °C min^−1^, and change in the millidegree ellipticity was monitored at 222 nm. After blank correction, an averaged normalized data of three independent experiments in the far UV range were considered for *T*_m_ calculation and fitting in a two-state transition model to obtain thermodynamic parameters.^34,38,39^

### 4.4 Limited proteolysis and glutaraldehyde crosslinking of BARD1 BRCT wild-type and mutant proteins

Initially, 2 mg mL^−1^ BARD1 BRCT wild-type and mutant proteins were incubated with 10 pg μL^−1^ trypsin and chymotrypsin (final concentration) each at 37 °C and 25 °C, respectively, for different time intervals of 0, 5, 10, 30, 60, 120 and 180 min. After incubation, the reaction was terminated individually by adding 1 mM PMSF (Sigma Aldrich), and the samples were heated after adding an equal volume of Laemmli buffer, and then analyzed over SDS-PAGE. Untreated BARD1 BRCT wild-type and mutant proteins with trypsin and chymotrypsin were considered as control, and experiments were performed in triplicates. Furthermore, wild-type and mutant proteins Cys645Arg, Val695Leu and Ser761Asn in buffer (10 mM HEPES, 150 mM NaCl, pH 7.5) were incubated with freshly prepared solution of glutaraldehyde (final concentration, 0.1%) for 0, 1, 2.5, 5, 10, 15, 30 and 60 minutes at 37 °C. The crosslinking reaction was terminated by adding 5 μL of 1 M Tris–HCl of pH 8.0. The cross-linked samples were heated after adding an equal volume of Laemmli sample buffer before loading and analysis on 12% SDS-PAGE.

### 4.5 Dynamic light scattering experiment (DLS) of BARD1 BRCT wild-type and mutant proteins

BARD1 BRCT wild-type and Cys645Arg, Val695Leu and Ser761Asn mutant proteins at a concentration of 1 mg mL^−1^ in buffer (10 mM HEPES, 150 mM NaCl, pH 7.5) were degassed and filtered (0.22 μM filter, Millipore) before subjecting to hydrodynamic analysis using a Malvern particle size analyzer (Zetasizer μV). All the proteins were scanned at 5 min intervals for 15 min. The experiment was repeated thrice and an average was considered to be the true value for calculating the effective diameter.

### 4.6 *In silico* prediction of BARD1 BRCT mutants

The possible pathogenic consequences of substitution mutations of BARD1 BRCT Cys645Arg, Val695Leu and Ser761Asn were studied using I-mutant 3.0,^32^ AGVGD^52,53^ and SNP&GO^33^ software tools.

### 4.7 Molecular dynamics simulation

Molecular dynamics simulation was performed for BARD1 BRCT and mutant structure with GROMACS 4.5.5 (ref. 54 and 55) using OPLS force field.^56^ Coordinates of BARD1 BRCT for MD simulation were taken from PDB ID: 2NTE.^30^ The systems were embedded in the simple point charge (SPC) water model, with a margin of 1.5 nm in between the protein and the boundaries of the periodic cubic box. The ionization state of the residue was set to be consistent with neutral pH and counter ions were added to neutralize the system. All the bond lengths were constrained using the SHAKE algorithm^57^ and electrostatic interactions were calculated by the particle mesh Ewald (PME) method.^58,59^ The solvated system was then subjected to energy minimization, as reported earlier,^34,60^ using the steepest-descent algorithm to eliminate any bad contacts with added water until a tolerance of 1000 kJ mol^−1^ was reached. The energy-minimized system was treated for equilibration by 100 ps NVT simulation at 300 K, followed by 100 ps of NPT simulation to achieve proper equilibration of the simulated system. Final production simulations were performed in the isothermal isobaric (NPT) ensemble at 300 K using an external bath with a coupling constant of 0.1 ps. The pressure was kept constant (1 bar) using pressure coupled with the time constant set to 1 ps. The trajectories were stored at every 2 ps during the simulation. Analysis of RMSD, RMSF, radius of gyration, number of hydrogen bonds, molecular volume and SASA were performed using the GROMACS 4.5.5 inbuilt commands.

### 4.8 Principal component analysis (PCA)

PCA was preformed over the wild-type and mutant protein trajectories. Eigenvalues and eigenvectors were calculated by diagonalizing the covariance matrix. Furthermore, covariance analysis was performed for the BARD1 BRCT wild-type and Cys645Arg, Val695Leu and Ser761Asn mutant proteins with Gromacs 4.5.5. Cross-correlation for PCA was performed on the Cα-atoms of the respective trajectories using the Prody software.^61^

## Conflicts of interest

All the authors declare that is no conflict of interest.

## Supplementary Material

RA-008-C8RA06524A-s001

## References

[cit1] Baer R., Ludwig T. (2002). Curr. Opin. Genet. Dev..

[cit2] Brzovic P. S., Meza J. E., King M. C., Klevit R. E. (2001). J. Biol. Chem..

[cit3] Brzovic P. S., Rajagopal P., Hoyt D. W., King M. C., Klevit R. E. (2001). Nat. Struct. Biol..

[cit4] Baer R., Ludwig T. (2002). Curr. Opin. Genet. Dev..

[cit5] Hashizume R., Fukuda M., Maeda I., Nishikawa H., Oyake D., Yabuki Y., Ogata H., Ohta T. (2001). J. Biol. Chem..

[cit6] Greenberg R. A., Sobhian B., Pathania S., Cantor S. B., Nakatani Y., Livingston D. M. (2006). Genes Dev..

[cit7] Brzovic P. S., Keeffe J. R., Nishikawa H., Miyamoto K., Fox D., Fukuda M., Ohta T., Klevit R. (2003). Proc. Natl. Acad. Sci. U. S. A..

[cit8] Xia Y., Pao G. M., Chen H.-W., Verma I. M., Hunter T. (2003). J. Biol. Chem..

[cit9] Chen C. F., Chen L. W., Chien C. T., Wu M. S., Tsai T. J. (1996). Clin. Exp. Pharmacol. Physiol..

[cit10] Feki A., Jefford C. E., Durand P., Harb J., Lucas H., Krause K. H., Irminger-Finger I. (2004). Biol. Reprod..

[cit11] Rodriguez J. A., Schuchner S., Au W. W., Fabbro M., Henderson B. R. (2004). Oncogene.

[cit12] Rodriguez J. A., Au W. W., Henderson B. R. (2004). Exp. Cell Res..

[cit13] Cohen M., Meisser A., Haenggeli L., Irminger-Finger I., Bischof P. (2007). Mol. Hum. Reprod..

[cit14] Wu Q., Paul A., Su D., Mehmood S., Foo T. K., Ochi T., Bunting E. L., Xia B., Robinson C. V., Wang B. (2016). Mol. Cell.

[cit15] Thai T. H., Du F., Tsan J. T., Jin Y., Phung A., Spillman M. A., Massa H. F., Muller C. Y., Ashfaq R., Mathis J. M., Miller D. S., Trask B. J., Baer R., Bowcock A. M. (1998). Hum. Mol. Genet..

[cit16] Fox, 3rd D., Le Trong I., Rajagopal P., Brzovic P. S., Stenkamp R. E., Klevit R. E. (2008). J. Biol. Chem..

[cit17] Glover J. M., Williams R. S., Lee M. S. (2004). Trends Biochem. Sci..

[cit18] Stucki M., Clapperton J. A., Mohammad D., Yaffe M. B., Smerdon S. J., Jackson S. P. (2005). Cell.

[cit19] Varma A. K., Brown R. S., Birrane G., Ladias J. A. (2005). Biochemistry.

[cit20] Leung C. C. Y., Glover J. M. (2011). Cell Cycle.

[cit21] Rappold I., Iwabuchi K., Date T., Chen J. (2001). J. Cell Biol..

[cit22] Manke I. A., Lowery D. M., Nguyen A., Yaffe M. B. (2003). Science.

[cit23] Masson M., Niedergang C., Schreiber V., Muller S., Menissier-de Murcia J., de Murcia G. (1998). Mol. Cell. Biol..

[cit24] Taylor R. M., Thistlethwaite A., Caldecott K. W. (2002). Mol. Cell. Biol..

[cit25] Thanassoulas A., Nomikos M., Theodoridou M., Yannoukakos D., Mastellos D., Nounesis G. (2010). Biochim. Biophys. Acta.

[cit26] Li M., Yu X. (2013). Cancer Cell.

[cit27] Ishitobi M., Miyoshi Y., Hasegawa S., Egawa C., Tamaki Y., Monden M., Noguchi S. (2003). Cancer Lett..

[cit28] Ghimenti C., Sensi E., Presciuttini S., Brunetti I. M., Conte P., Bevilacqua G., Caligo M. A. (2002). Genes, Chromosomes Cancer.

[cit29] Laufer M., Nandula S. V., Modi A. P., Wang S., Jasin M., Murty V. V., Ludwig T., Baer R. (2007). J. Biol. Chem..

[cit30] Birrane G., Varma A. K., Soni A., Ladias J. A. (2007). Biochemistry.

[cit31] Tavtigian S. V., Greenblatt M. S., Lesueur F., Byrnes G. B. (2008). Hum. Mutat..

[cit32] Capriotti E., Fariselli P., Rossi I., Casadio R. (2008). BMC Bioinf..

[cit33] Calabrese R., Capriotti E., Fariselli P., Martelli P. L., Casadio R. (2009). Hum. Mutat..

[cit34] Choudhary R. K., Vikrant, Siddiqui Q. M., Thapa P. S., Raikundalia S., Gadewal N., Kumar N. S., Hosur M., Varma A. K. (2015). J. Biomol. Struct. Dyn..

[cit35] Ekblad C. M., Wilkinson H. R., Schymkowitz J. W., Rousseau F., Freund S. M., Itzhaki L. S. (2002). J. Mol. Biol..

[cit36] Ekblad C. M., Friedler A., Veprintsev D., Weinberg R. L., Itzhaki L. S. (2004). Protein Sci..

[cit37] Thanassoulas A., Nomikos M., Theodoridou M., Stavros P., Mastellos D., Nounesis G. (2011). Int. J. Biol. Macromol..

[cit38] Pace C. N., Shaw K. L. (2000). Proteins.

[cit39] Vikrant, Nakhwa P., Badgujar D. C., Kumar R., Rathore K. K., Varma A. K. (2014). J. Biomol. Struct. Dyn..

[cit40] Lakowicz J. R. , Principles of fluorescence spectroscopy, Springer Science & Business Media, 2013

[cit41] Guex N., Peitsch M. C. (1997). Electrophoresis.

[cit42] Pace C. N., Scholtz J. M. (1998). Biophys. J..

[cit43] Sternberg M. J., Chickos J. S. (1994). Protein Eng..

[cit44] Chou P. Y., Fasman G. D. (1978). Annu. Rev. Biochem..

[cit45] Hermans J. (1993). Curr. Opin. Struct. Biol..

[cit46] Creamer T. P., Rose G. D. (1994). Proteins: Struct., Funct., Bioinf..

[cit47] Coquelle N., Green R., Glover J. M. (2011). Biochemistry.

[cit48] Liu J., Pan Y., Ma B., Nussinov R. (2006). Structure.

[cit49] Li M., Lu L.-Y., Yang C.-Y., Wang S., Yu X. (2013). Genes Dev..

[cit50] Anisimov V. M., Ziemys A., Kizhake S., Yuan Z., Natarajan A., Cavasotto C. N. (2011). J. Comput.-Aided Mol. Des..

[cit51] You W., Yu-ming M. H., Kizhake S., Natarajan A., Chia-en A. C. (2016). PLoS Comput. Biol..

[cit52] Tavtigian S. V., Deffenbaugh A. M., Yin L., Judkins T., Scholl T., Samollow P. B., de Silva D., Zharkikh A., Thomas A. (2006). J. Med. Genet..

[cit53] Mathe E., Olivier M., Kato S., Ishioka C., Hainaut P., Tavtigian S. V. (2006). Nucleic Acids Res..

[cit54] Berendsen H., Grigera J., Straatsma T. (1987). J. Phys. Chem..

[cit55] Berendsen H. J., van der Spoel D., van Drunen R. (1995). Comput. Phys. Commun..

[cit56] Cornell W. D., Cieplak P., Bayly C. I., Gould I. R., Merz K. M., Ferguson D. M., Spellmeyer D. C., Fox T., Caldwell J. W., Kollman P. A. (1995). J. Am. Chem. Soc..

[cit57] Hess B., Kutzner C., Van Der Spoel D., Lindahl E. (2008). J. Chem. Theory Comput..

[cit58] Kholmurodov K., Smith W., Yasuoka K., Darden T., Ebisuzaki T. (2000). J. Comput. Chem..

[cit59] Cheatham T. I., Miller J., Fox T., Darden T., Kollman P. (1995). J. Am. Chem. Soc..

[cit60] Choudhary R. K., Siddiqui M. Q., Thapa P. S., Gadewal N., Nachimuthu S. K., Varma A. K. (2017). Sci. Rep..

[cit61] Bakan A., Meireles L. M., Bahar I. (2011). Bioinformatics.

