# Supplementary material for: Biophysical evaluation to categorize pathogenicity of cancer-predisposing mutations identified in the BARD1 BRCT domain
Source: RSC Adv. 2018 Oct 3;8(59):34056–68. doi: 10.1039/c8ra06524a (PMC9086705; doi:10.1039/c8ra06524a)

Electronic Supplementary Material (ESI) for RSC Advances.
This journal is © The Royal Society of Chemistry 2018

Legends to Supplementary figures: -

1. (**Figure 1 supplementary**): -**(A)** Structure of BARD1BRCT (PDB ID: **2NTE**) showing cancer - predisposing mutation sites. All the mutant sites are demarcated in red. **(B)** Expression and purification profile of BARD1BRCT *wild-type* and mutants. **(C)** Comparative overlay of elution profile of BARD1BRCT *wild-type* and mutant proteins in superdex-75 column (GE Healthcare).
2. **(Figure 2 supplementary): - Comparative chemical crosslinking analysis of BARD1BRCT *wild-type* and mutant proteins**. **(A)** Chemical crosslinking of BARD1BRCT *wild-type*, **(B)** Cys 645 Arg, **(C)** Ser 761 Asn and **(D)** Val 695 Leu. Control (**C**) shows untreated sample with glutaraldehyde.
3. **(Figure 3 Supplementary): - Comparative overlay of fluorescence emission spectra of BARD1BRCT *wild-type* and mutants to ascertain intermediate formation**. **(A)** Thermal denaturation of BARD1BRCT *wild-type***(B)** Val 695 Leu, **(C)** Ser 761 Asn and **(D)** Cys 645 Arg.
4. **(Figure 4 supplementary): - Comparative representation of *Wild-type* and mutant proteins showing average number of intra-molecular hydrogen bond formed during the MD simulation.** The average number of intra-molecular hydrogen bond is represented for every 10ns of MDS. *Wild-type* (Black), Cys 645 Arg (Green), Val 695 Leu (Blue) and Ser 761Asn (Red).
5. **(Figure 5 supplementary): - Comparative overlay of Eigenvalue on Eigenvector index for *wild-type* and mutant proteins.** Comparative profile of Eigenvalue attained for their respective Eigenvector for *wild-type* (black), Cys 645 Arg (green), Val 695 Leu (blue) and Ser 761 Asn (red) mutant proteins.
6. **(Figure 6 supplementary): - Representation of projections of first two Eigenvectors on Residues for *wild-type* and mutant proteins**: - **(A)** and **(B)** Projection of Eigenvector 1 and 2 on residue for *Wild-type*. **(C)** and **(D)** Projection of Eigenvector 1 and 2 on residue for Cys 645 Arg. **(E)** and **(F)** Projection of Eigenvector 1 and 2 on residue for Val 695 Leu. **(G)** and **(H)** Projection of Eigenvector 1 and 2 on residue for Ser 761 Asn.
7. **(Figure 7 supplementary): - Representative RMSF structures of *wild-type* and mutant proteins.** The most flexible region is represented in red and rigid regions during the MDS are in blue. **(A)** *Wild-type*, **(B)** Cys 645 Arg, **(C)** Val 695 Leu and **(D)** Ser 761 Asn.
8. **(Figure 8 supplementary): - Comparative secondary structure representation of *Wild-type* and mutant proteins showing different components of secondary structure. 4 (A)** Secondary structure of *wild-type* protein, **(B)** Cys 645 Arg,**(C)** Val 695 Leu and **(D)** Ser 761 Asn**.**
9. **(Figure 9 Supplementary): - Comparative analysis of Intra-molecular interaction of *wild–type* and mutant protein: - (A)** BARD1BRCT *Wild-type* and **(B)** Cys 645 Arg, **(C)** BARD1BRCT *wild-type* and **(D)** Val 695 Leu, **(E)** BARD1BRCT *wild-type* and **(F)** Ser 761 Asn

Supplementary Figure-1

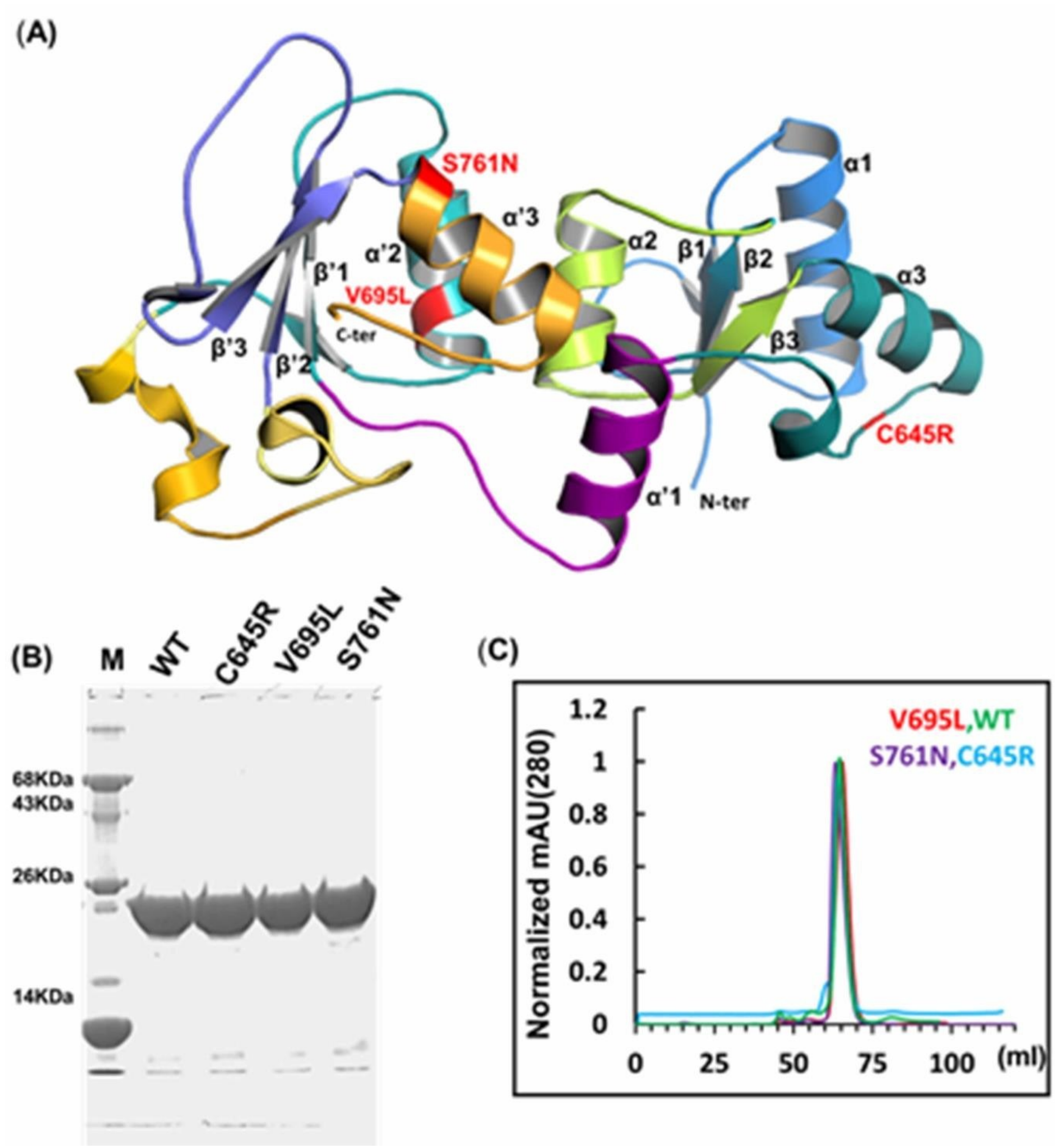

Supplementary Figure-2

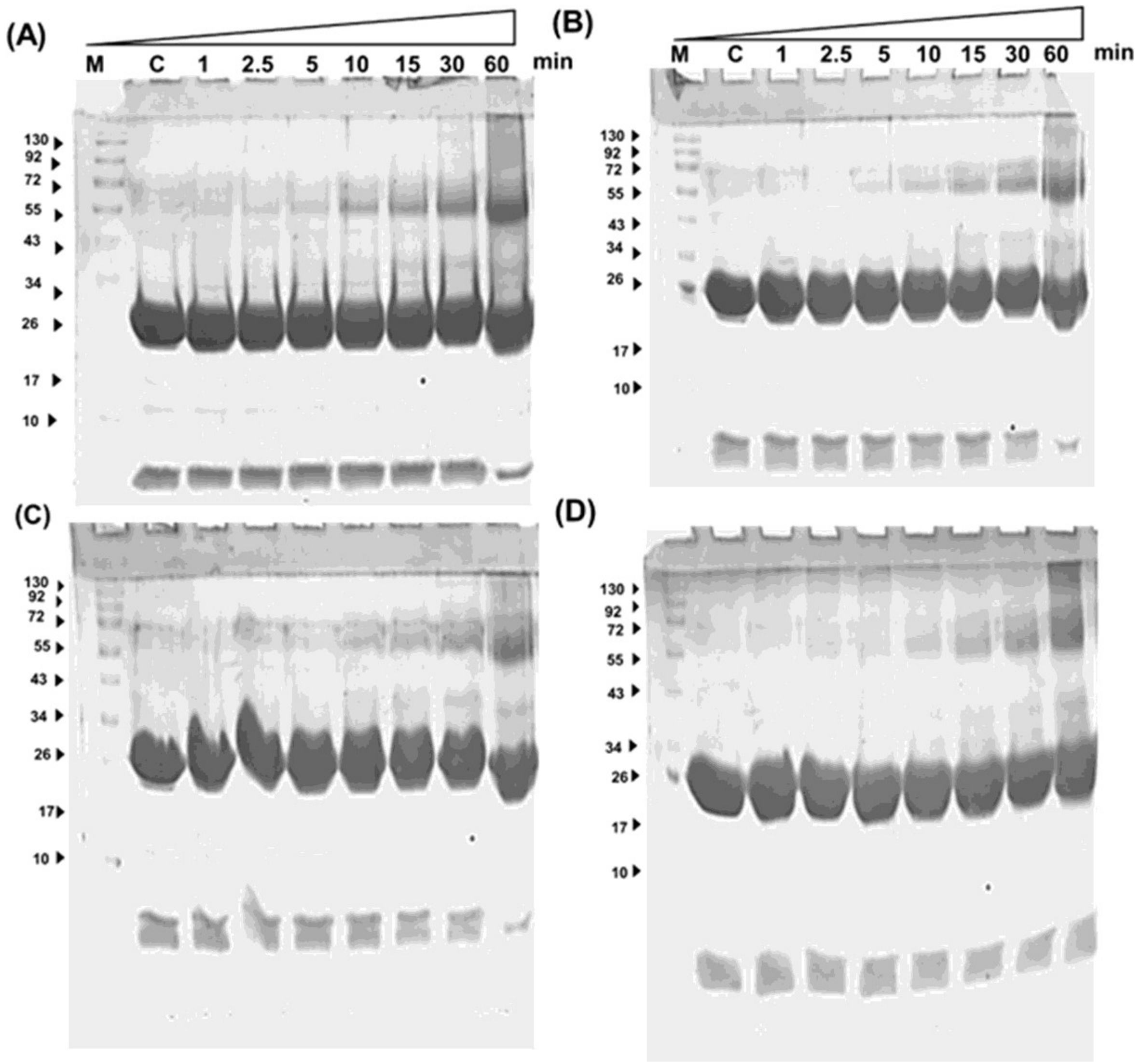

Supplementary Figure-3

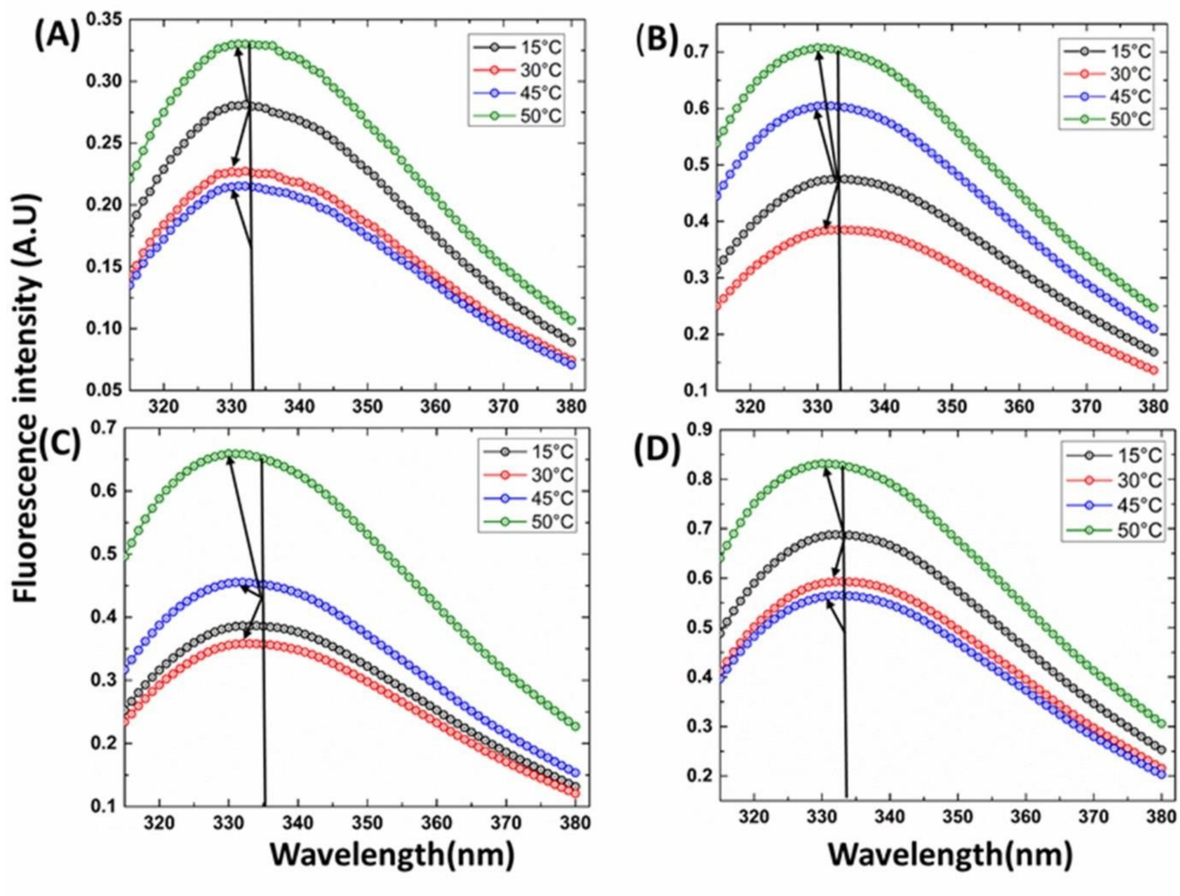

Supplementary Figure -4

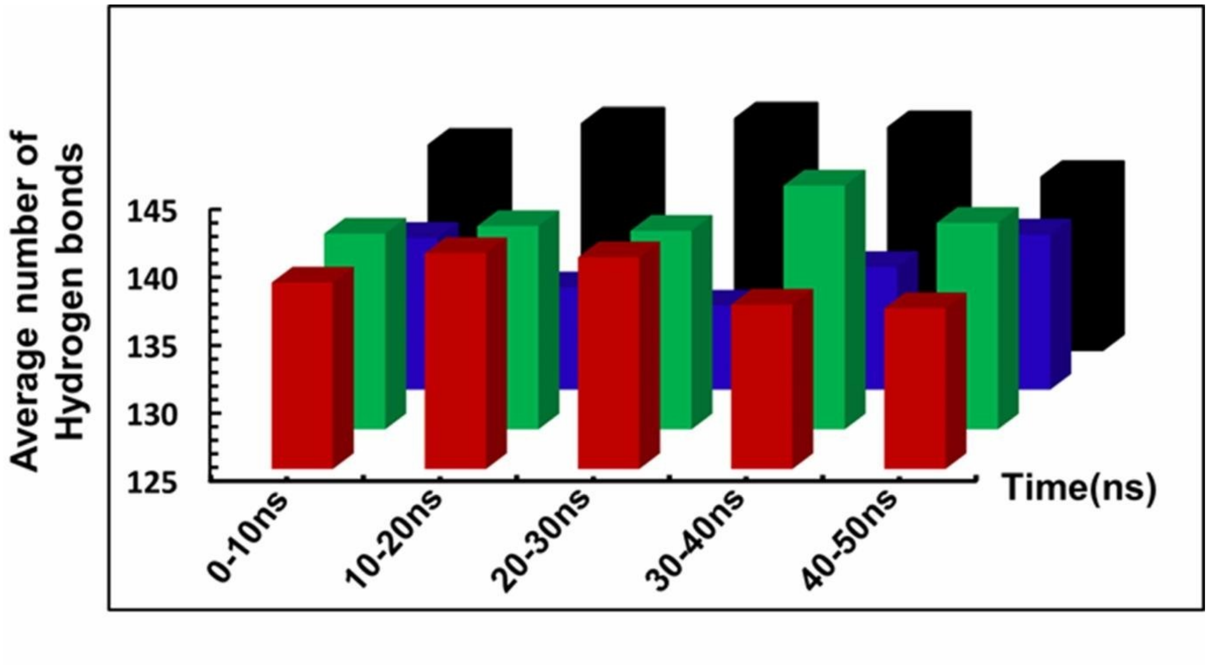

Supplementary Figure -5

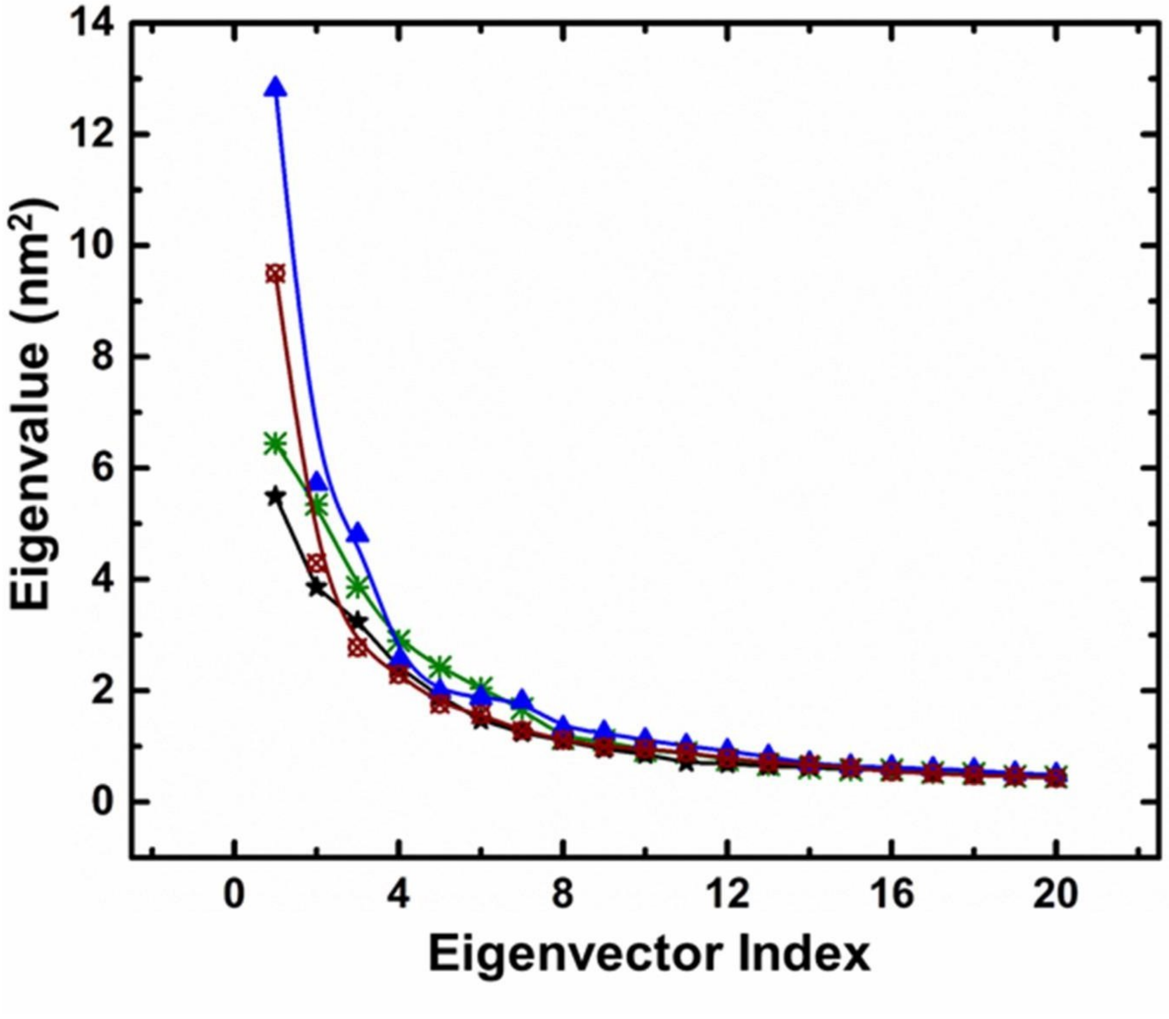

Supplementary Figure-6

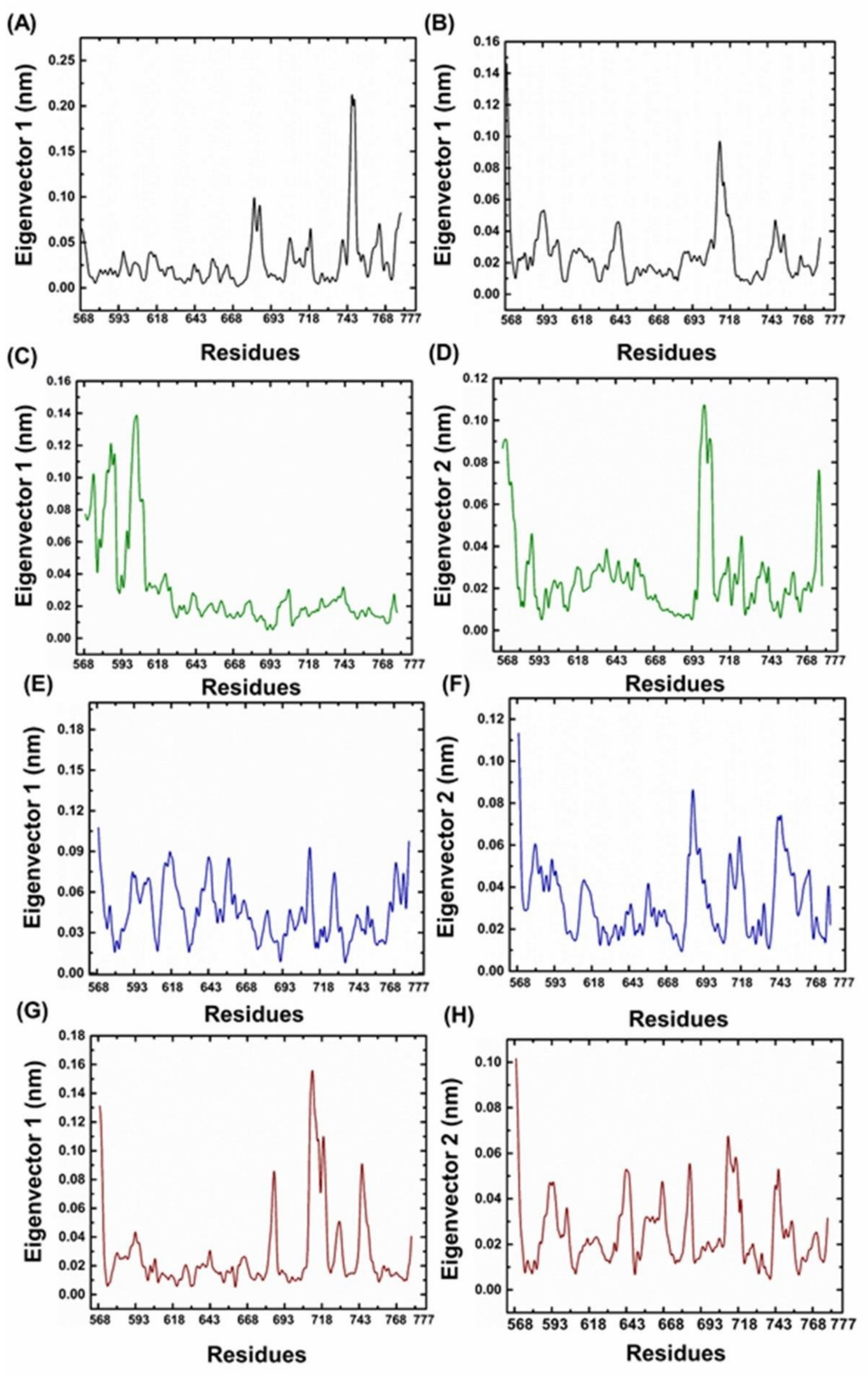

Supplementary Figure-7

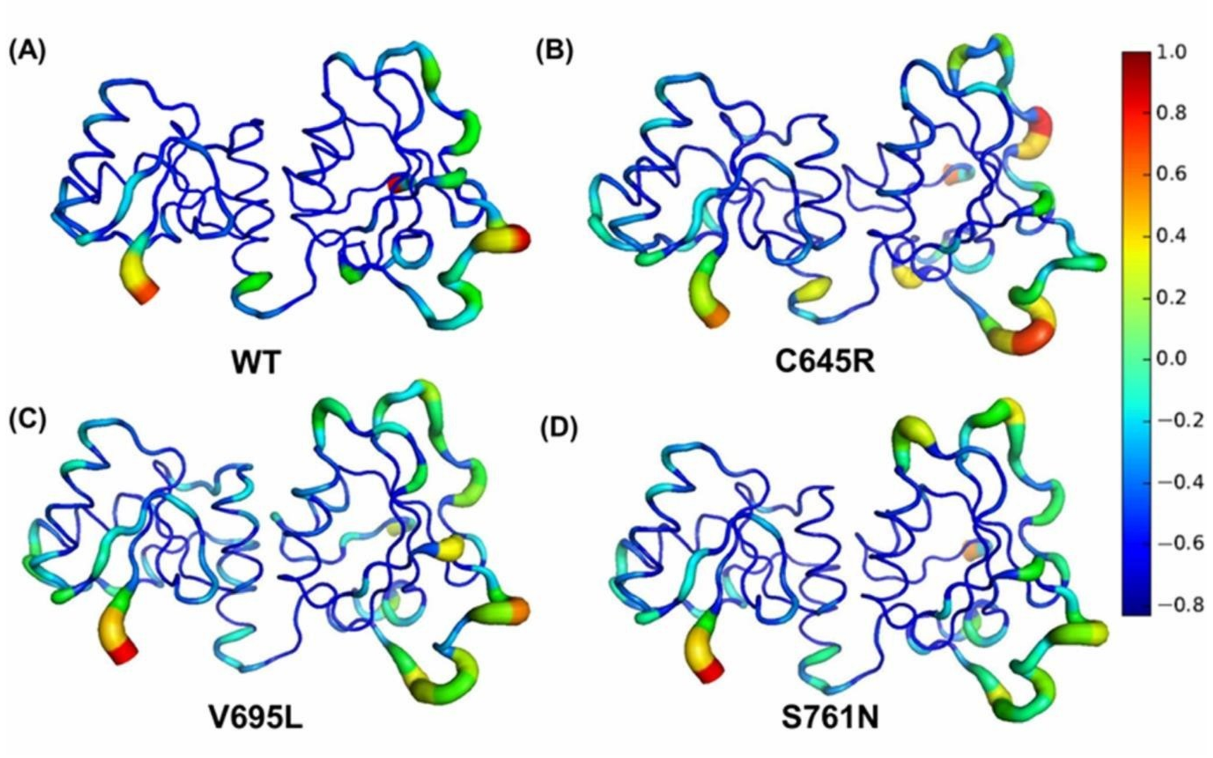

Supplementary Figure -8

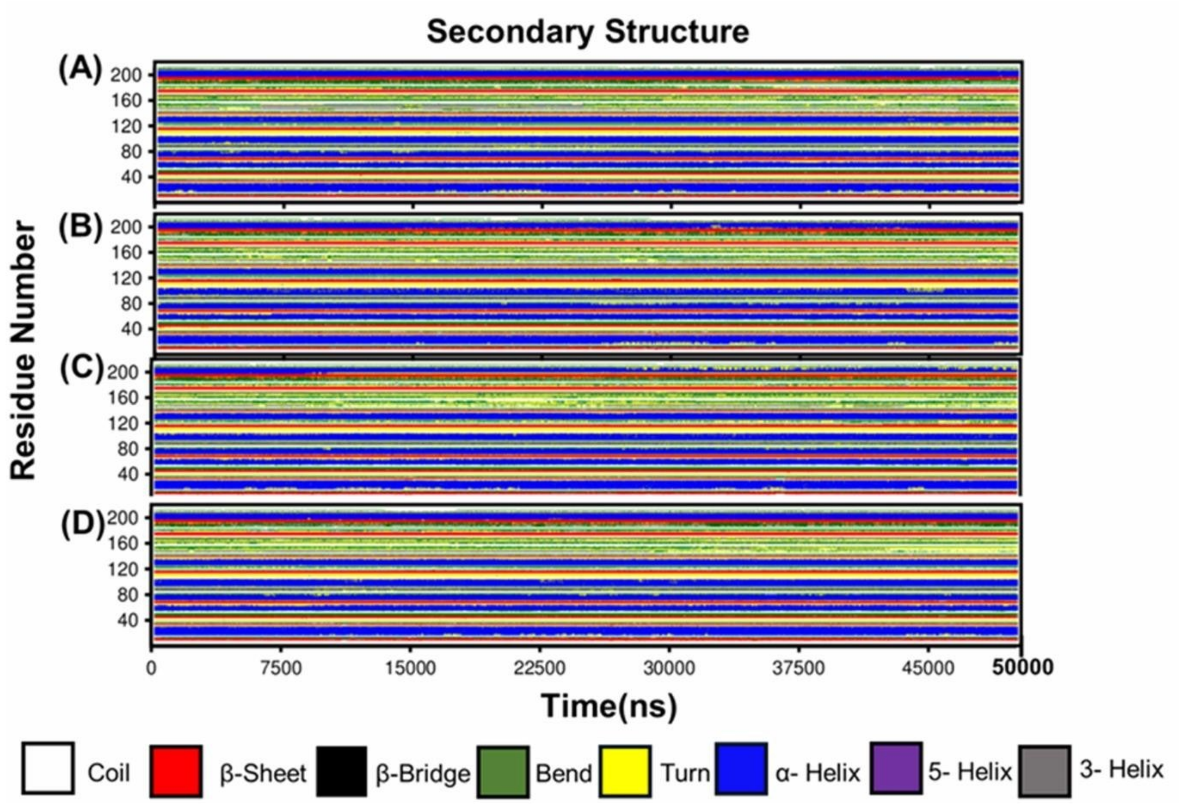

Supplementary Figure-9

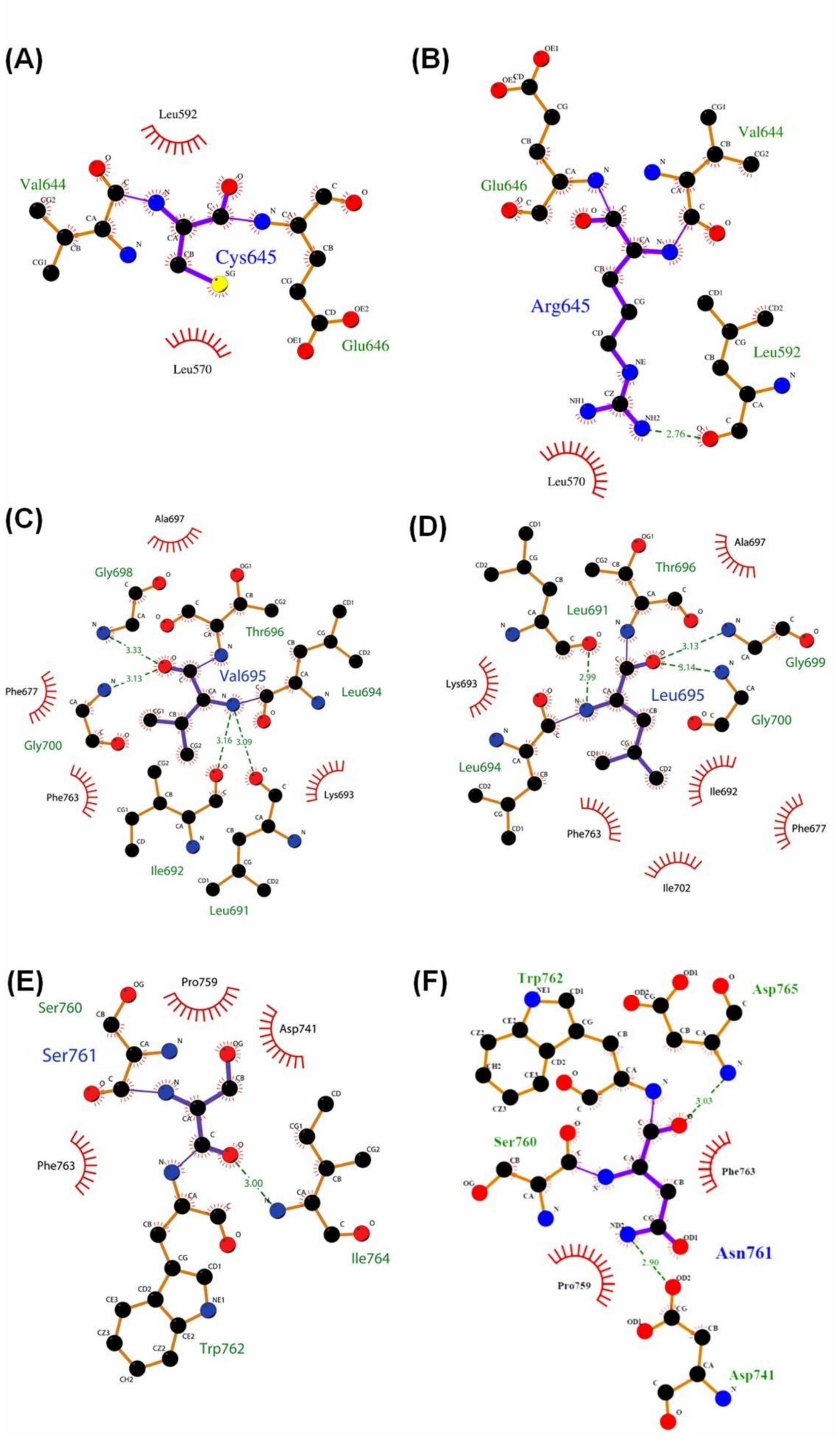

Supplement: RA-008-C8RA06524A-s001 [file RA-008-C8RA06524A-s001.pdf]
